# Evolutionary History of Rabies in Ghana

**DOI:** 10.1371/journal.pntd.0001001

**Published:** 2011-04-05

**Authors:** David T. S. Hayman, Nicholas Johnson, Daniel L. Horton, Jessica Hedge, Philip R. Wakeley, Ashley C. Banyard, Shoufeng Zhang, Andy Alhassan, Anthony R. Fooks

**Affiliations:** 1 Rabies and Wildlife Zoonoses Group, Veterinary Laboratories Agency – Weybridge, Surrey, United Kingdom; 2 Cambridge Infectious Diseases Consortium, Department of Veterinary Medicine, University of Cambridge, Cambridge, United Kingdom; 3 Institute of Zoology, Zoological Society of London, Regent's Park, London, United Kingdom; 4 Institute for Evolutionary Biology, University of Edinburgh, Edinburgh, United Kingdom; 5 Veterinary Services Laboratory, Ministry of Food and Agriculture, Accra, Ghana; 6 Changchun Veterinary Research Institute, Chinese Academy of Agricultural Science, Changchun, Jilin, People's Republic of China; 7 The National Centre for Zoonosis Research, University of Liverpool, Leahurst, Neston, United Kingdom; Swiss Tropical Institute, Switzerland

## Abstract

Rabies virus (RABV) is enzootic throughout Africa, with the domestic dog (*Canis familiaris*) being the principal vector. Dog rabies is estimated to cause 24,000 human deaths per year in Africa, however, this estimate is still considered to be conservative. Two sub-Saharan African RABV lineages have been detected in West Africa. Lineage 2 is present throughout West Africa, whereas Africa 1a dominates in northern and eastern Africa, but has been detected in Nigeria and Gabon, and Africa 1b was previously absent from West Africa. We confirmed the presence of RABV in a cohort of 76 brain samples obtained from rabid animals in Ghana collected over an eighteen-month period (2007–2009). Phylogenetic analysis of the sequences obtained confirmed all viruses to be RABV, belonging to lineages previously detected in sub-Saharan Africa. However, unlike earlier reported studies that suggested a single lineage (Africa 2) circulates in West Africa, we identified viruses belonging to the Africa 2 lineage and both Africa 1 (a and b) sub-lineages. Phylogeographic Bayesian Markov chain Monte Carlo analysis of a 405 bp fragment of the RABV nucleoprotein gene from the 76 new sequences derived from Ghanaian animals suggest that within the Africa 2 lineage three clades co-circulate with their origins in other West African countries. Africa 1a is probably a western extension of a clade circulating in central Africa and the Africa 1b virus a probable recent introduction from eastern Africa. We also developed and tested a novel reverse-transcription loop-mediated isothermal amplification (RT-LAMP) assay for the detection of RABV in African laboratories. This RT-LAMP was shown to detect both Africa 1 and 2 viruses, including its adaptation to a lateral flow device format for product visualization. These data suggest that RABV epidemiology is more complex than previously thought in West Africa and that there have been repeated introductions of RABV into Ghana. This analysis highlights the potential problems of individual developing nations implementing rabies control programmes in the absence of a regional programme.

## Author Summary

Rabies virus (RABV) is widespread throughout Africa, with the domestic dog being the principal vector. Dog rabies is estimated to cause 24,000 human deaths per year in Africa, however, this estimate is still considered to be conservative. Two sub-Saharan African RABV lineages (Africa 1 and 2) are thought to circulate in western and central Africa. We confirmed the presence of RABV in a cohort of 76 brain samples obtained from rabid animals in Ghana collected from 2007 to 2009. In addition we developed and tested a novel molecular diagnostic assay for the detection of RABV, which offers an alternative RABV diagnostic tool for African laboratories. Our analysis of the genetic sequences obtained confirmed all viruses to be RABV, however, unlike previous studies we detected two sub-Saharan African RABV viruses (Africa 1 and 2) in this cohort, which included a single virus previously undetected in West Africa. We suggest that there has been repeated introduction of new RABVs into Ghana over a prolonged period from other West African countries and more recently from eastern Africa. These observations further highlight the problems of individual developing nations implementing rabies control programmes at a local, rather than regional level.

## Introduction

Viruses belonging to the genus *Lyssavirus*, family *Rhabdoviridae*, cause the disease rabies. Rabies virus (RABV) is enzootic throughout Africa with the domestic dog (*Canis familiaris*) being the principal vector [1]. Sylvatic rabies is also reported in a number of wildlife hosts, particularly in southern Africa [2], [3], [4], [5]. Rabies remains the only disease known to have a 100% mortality rate and has a high DALY (disability adjusted life years) score compared with other ‘neglected zoonoses’ [1], [6], [7]. Dog rabies is estimated to cause 24,000 (7000–46000, 95% percentiles) human deaths per year in Africa [1], however, this figure is still considered to be a conservative estimate as rabies cases in humans are widely under-reported in parts of Africa [8], [9].

Rabies has been present within the dog population of Ghana for decades [10], [11]. Previously, control methods including dog vaccination and stray dog removal have been intermittent and not sustained. Unfortunately, as in several other developing African countries, rabies diagnostics within the Ghanaian veterinary services remains limited to non-*Lyssavirus* species specific staining techniques, including the Sellers' stain and fluorescent antibody test (FAT) [12]. Currently, only individual owners vaccinate their dogs for their (owner and dog) protection. Between 1970 and 1974, an average of 72 cases of canine rabies were reported annually throughout the country [10]. Between 1977 and 1981 this number increased to over 100 cases annually, with an incidence of human rabies cases rising to 27 in 1981 [11]. Since 1981 there have been no further published reports of rabies in Ghana, and rabies viruses from the country have not been included in phylogenetic analyses of rabies in Africa [13], [14]. The virus is believed to cause disease in approximately 0–60% of those patients that are exposed depending on route of exposure [8]. Despite this, 123 clinically-confirmed human cases were recorded by public health officials between 2000 and 2004 (unpublished results). Moreover, ‘suspect’ human rabies cases are rarely confirmed using a laboratory-based diagnosis, relying solely on a clinical diagnosis [9].

The first phylogenetic study of rabies viruses from sub-Saharan Africa established three genetically distinct lineages (Africa 1, 2, and 3) [15]. Sub-lineage Africa 1a dominates northern and eastern Africa, but has also been detected in Nigeria, Gabon and Madagascar, suggesting a very broad distribution. Sub-lineage 1b is found in eastern, central and southern Africa and lineage 2 is present in an uninterrupted band across West Africa as far east as Chad [13], [16]. Africa 1 and 2 lineages have been detected in a range of domestic and wild carnivore species. While domestic dogs appear to be the only population essential for maintenance of canid variants in some parts of Africa [17], [18], wild canids have been suggested to contribute to sustaining canine rabies cycles in specific geographic loci in South Africa and Zimbabwe [19], [20], [21]. A third lineage (Africa 3) is thought to be maintained within viverrid species in southern Africa [22], [23], [24]. This phylogenetic distinction has been supported by studies investigating rabies across Africa [13], [25], epidemiological studies of rabies within specific countries [3], [16], [18], [26], studies on wildlife populations [5], [27], [28] and investigations into the origin of human rabies [29], [30]. More recently another distinct lineage, Africa 4, has been identified in northern Africa [31].

The principal objectives of this study were to characterise the lyssaviruses causing rabies in Ghana and to understand the evolutionary history of the circulating viruses. We also assessed the performance of a novel isothermal amplification technique for the detection of rabies virus for use in African laboratories. The low threshold of technology required to use this technique for diagnosis of animal diseases in Africa has been advocated [32], [33].

## Methods

**Figure 1:**
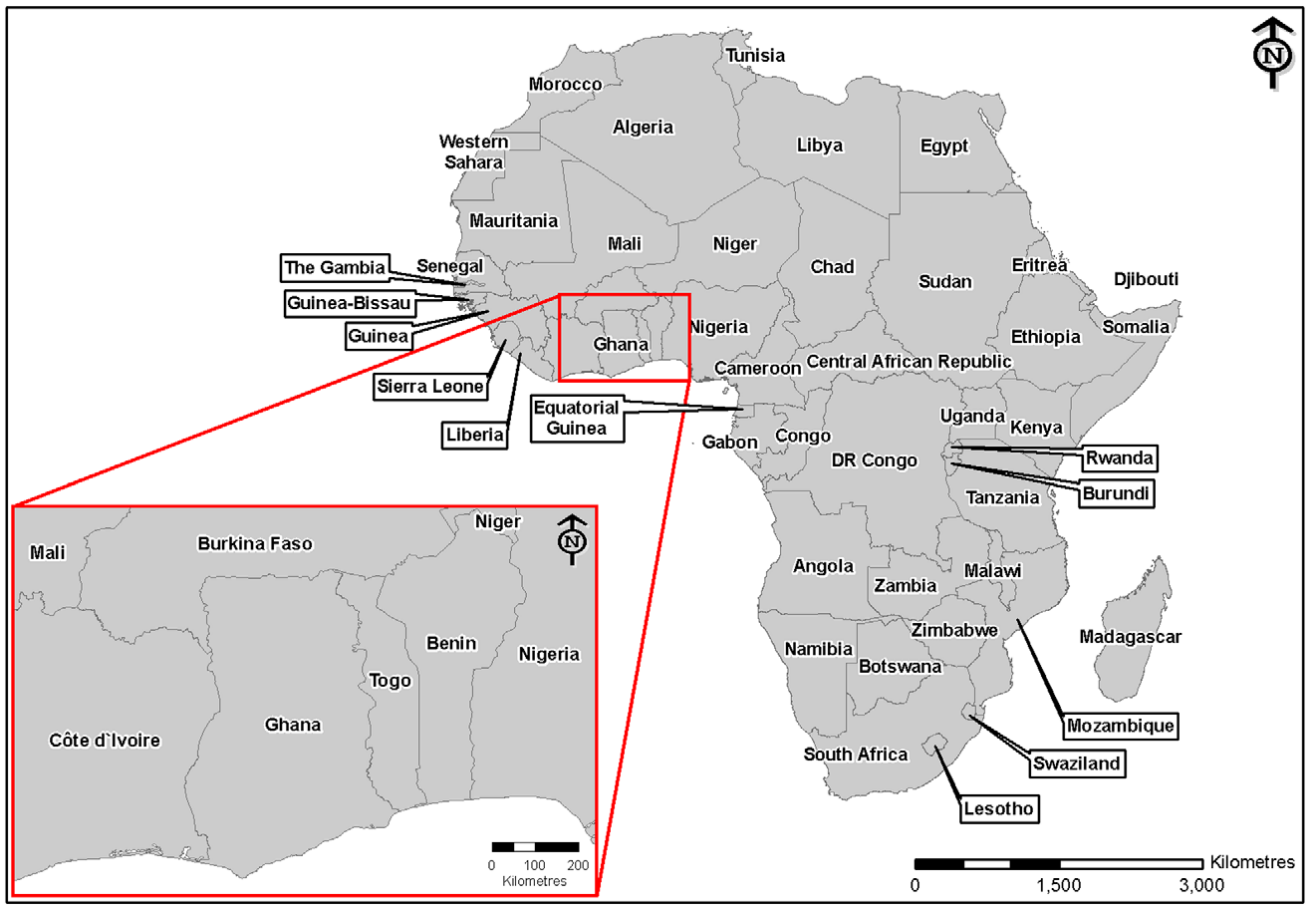
Map of Africa showing the location of Ghana.

The Republic of Ghana is on the southern coast of West Africa (Figure 1). It shares borders with Togo (east), Ivory Coast (west), and Burkina Faso (north). Ghana has several ecosystems broadly attributed to the patterns of rainfall and geological topology [34]. The south eastern coastline consists of mostly low plains and scrubland, and separates the upper and lower Guinea African forest systems. Southwest and south central Ghana is a semi-deciduous forested plateau. Savannah dominates the northern part of the country. There are geographical features that may represent barriers to rabies spread in Ghana. The highest point in Ghana is only 885 m above sea level along the eastern border, however, the world's largest artificial lake, Lake Volta, separates much of eastern Ghana from the rest [34]. Ghana's population has rapidly increased in the last few decades. A census in 1961 recorded 6.7 million people, however, the current estimate is approximately 24 million [35].

Brain samples were derived from dogs (74) and cats (2) brought to the central diagnostic veterinary laboratory (Veterinary Services Laboratory, VSL) in the capital of Ghana, Accra, on suspicion of being rabid (Table S1). The samples used in this study were obtained by the Ghanaian government's veterinary services laboratory from naturally infected rabid animals in Ghana. No samples were obtained from, nor animals used in, an experimental study. All samples were obtained from animals within 142 km of Accra. Infection with RABV was suspected from clinical signs and from test results using either Sellers' staining (*n = 69*) of Negri bodies or the FAT (*n = 7*) in the VSL [12]. The panel were assigned numbers randomly and transferred from the VSL to the Veterinary Laboratories Agency (VLA), Weybridge, UK, where further molecular analysis was undertaken.

Total RNA was extracted from each brain sample using Trizol (Invitrogen) following the manufacturer's protocol. Pellets were resuspended in 10 µL of HPLC grade water. Reverse transcription and polymerase chain reaction were performed using previously published methods to amplify a 600 bp region of the nucleoprotein gene [36].

A novel reverse-transcription loop-mediated isothermal amplification assay (RT-LAMP) was applied to a limited panel of ten samples systematically taken from the larger randomly numbered Ghanaian panel. Previous reports applied this technique to viruses from a range of countries [37], [38] or to fixed rabies virus [32]. The assay is composed of two sets of primers (Table 1). The first, designated Rab1, amplifies viruses belonging to the cosmopolitan lineage. The second, Rab4, amplifies viruses belonging to the arctic lineage. A reaction mixture incorporating a combination of all 12 primers amplifies viruses from both groups (data not shown). 1 µg of each RNA sample was added to a reaction mixture containing each of the 12 primers at the final concentration indicated in Table 1, Isothermal Mastermix (GeneSys Ltd) and 0.12 units Thermoscript reverse transcriptase (RT) (Invitrogen) in a final reaction volume of 25 µl. A cosmopolitan RABV obtained from a Turkish dog that had been used to develop the assay (data not shown) was included as a positive control. A no-template control sample (HPLC grade water) was used as a negative control. The reaction was incubated at 65°C for 1 hour. A 10 µl aliquot was removed and mixed with 2 µl sample loading buffer and loaded onto a 1% agarose gel containing ethidium bromide and separated at 80 volts for 1 hour. The amplification products were visualized by UV irradiation. The RT-LAMP assay was further adapted for use with a lateral flow device (LFD) for visualization of RT-LAMP products. The assay was run with the above conditions and reagents, but with the alternative loop primer sets (Table 1: Rab1 FLOOPFlc, Rab1 BLOOPBtn, Rab3 FLOOPFlc, Rab3 BLOOPBtn, Forsite Diagnostics). The LFD (Forsite) uses a mouse anti-biotin monoclonal antibody (MAb) in the “get wet” strip to indicate the LFD run succeeded and a mouse anti-fluorescein MAb to bind the LAMP product to the fluorescein tag to show a positive result. The product was diluted in 1∶500 volumes of HPLC grade water and 60 µl added to the LFD test well.

**Table 1:** Reverse-transcription loop-mediated isothermal amplification primers used in this study.

Assay	Description	Primer Name	Primer Sequence (5′-3′)	Final concentration
Rab1	Outer primers	Rab1 F3	AGCCCCCGACTTAAACAAAG	5 pmoles
		Rab1 B3	CTGTCAGAGCCCAATTTCCT	5 pmoles
	Inner primers	Rab1 FIP	GCATTGCTGCTGCCAAGTAGGATTTTCAGGCATGAATGCAGCCA	50 pmoles
		Rab1 BIP	CGTGTCCAGAAGACTGGACCAGTTTTATTTCCACCAGAGAATCC	50 pmoles
	Loop primers	Rab1 FLOOP	ACATACATCATCAGGATCAAGT	25 pmoles
		Rab1 BLOOP	CTATGGAATCTTGATCGCACG	25 pmoles
Rab4	Outer primers	Rab4 F3	GCCCCCGATTTGAACAA	5 pmoles
		Rab4 B3	GGGAATTGGGCTTTGACG	5 pmoles
	Inner primers	Rab4 FIP	ACTGCATCGCAGCTGCTAAGTAGGATTTTCAGGCTTGAATGCTGCCAA	50 pmoles
		Rab4 BIP	CATGTCCTGAAGACTGGACCAGTTTTATCTCCACAAGAGAATCTGGGGT	50 pmoles
	Loop primers	Rab4 FLOOP	ACATACATCAGGATCAAGC	25 pmoles
		Rab4 BLOOP	CTATGGGATCTTGATTGCAAG	25 pmoles
LFD	Loop primers	Rab1 FLOOPFlc	ACATACATCATCAGGATCAAGT	25 pmoles
		Rab1 BLOOPBtn	{Btn}CTATGGAATCTTGATCGCACG	25 pmoles
		Rab3 FLOOPFlc	{Flc}ACATACATCAGGATCAAGC	25 pmoles
		Rab3 BLOOPBtn	{Btn}CTATGGGATCTTGATTGCAAG	25 pmoles

Flc Fluorescein.

Btn Biotin.

Direct consensus DNA sequencing of a 405 bp region of the nucleoprotein (N) gene was undertaken as previously described [39]. Sequences produced were edited using SeqMan (DNAstar Lasergene) and aligned (ClustalW, Megalign, DNAstar Lasergene). Further analysis of the newly derived sequences was undertaken using Bayesian Markov chain Monte Carlo (MCMC) phylogenetic analysis using BEAST software (version 1.6.1) [40] with a panel of pan-African RABV selected from GenBank (Table S2). Sequences were aligned in ClustalX2 (version 1.2). A relaxed-clock (uncorrelated lognormal) [41] was employed in conjunction with a general time reversible (GTR) model of substitution with gamma distributed variation in rates amongst sites and a proportion of sites assumed to be invariant. This method allows the evolutionary rate of each branch to vary without assuming these rates are correlated among adjacent branches. A model of constant population size was employed for the phylogeographic analysis, motivated by a preliminary analysis of the data using a non-parametric model of growth under which suggested no significant deviation from the constant size. The MCMC was run for 30,000,000 steps with parameters and trees sampled every 6,000 steps. Parameter effective sample sizes were >100 and posterior distributions were inspected to ensure adequate mixing in Tracer (version 1.5). A phylogeographic approach was not taken to analyze the correlation between lineage and distance, due to all animals reportedly originating within close proximity from central Accra. To infer the temporal and spatial diffusion of Africa 1 and Africa 2 clades into Ghana, a continuous-time Markov chain (CTMC) process over discrete sampling locations was employed in a phylogeographic analysis of each clade using BEAST. The sampling origin for each sequence was considered to be the centroid of the country from which the sequence was sampled [14]. The same models of nucleotide substitution, growth and clock rate were employed as before, but an MCMC chain length of 100 million steps was used to ensure sufficient mixing and convergence of all phylogeographic parameters, and trees were logged every 20,000 steps. An appropriate (maximum 10%) burn-in was removed from each and the sampled trees were summarized as maximum clade credibility (MCC) trees. All sequences reported in this study (Table S1) were deposited in GenBank.

## Results

**Figure 2:**
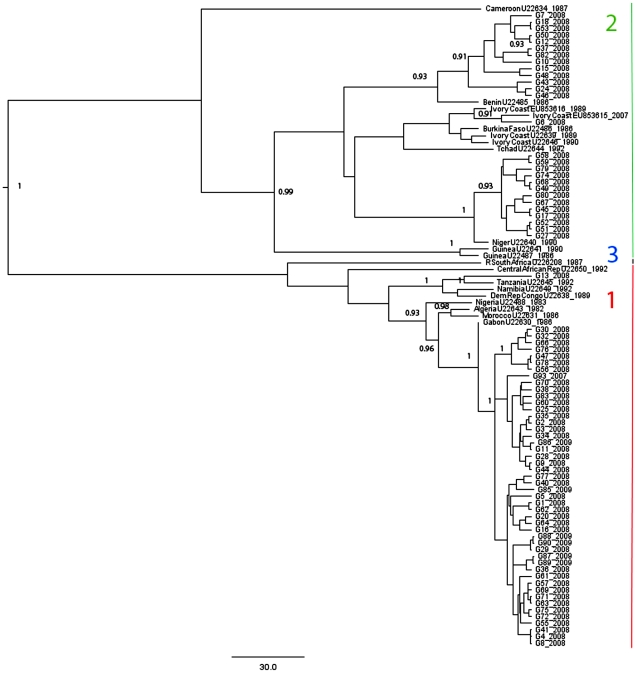
A maximum clade credibility phylogenetic tree of African rabies viruses (RABV). 405base-pair regions of the RABV nucleoprotein from viruses derived from Ghanaian samples (prefixed G) and selected RABV from GenBank were analyzed. Posterior values greater than 0.9 are shown. African RABV lineages 1, 2 and 3 are labelled on the right-hand side. The scale bar represents 30 years.

Seven of the 69 samples from suspected rabies cases tested at the VSL Accra were negative by Sellers' stain, whereas each of the seven tested by FAT was positive. Due to clinical signs exhibited by the animals, all 76 samples were included for further analysis at VLA-Weybridge and were subsequently positive by RT-PCR for RABV. Sequence analysis demonstrated that all viruses belonged to lineages previously reported from Africa. Twenty-seven samples were from the Africa 2 lineage, 48 samples from the Africa 1a sub-lineage, and a solitary sequence (sample G13) belonged to the Africa 1b sub-lineage (Table S1, Figure 2).

The MCMC tree of a 405 bp region of the 76 RABV N gene sequences analyzed with 20 African RABV sequences from GenBank is shown in Figure 2. The topology is similar to other analyses of African RABV N genes [15] that included Africa 1, 2, and 3 lineages. Rabies viruses from Ghana clearly form two lineages, Africa 1 (49 viruses) and 2 (27 viruses). Within each lineage sequences are separated into sub-lineages, in the case of Africa 1, or clades in that of the Africa 2 lineage. Our analysis estimates that the Africa 2 lineage diverged approximately 181 years ago (73–313 yrs, 95% HPD).

**Figure 3:**
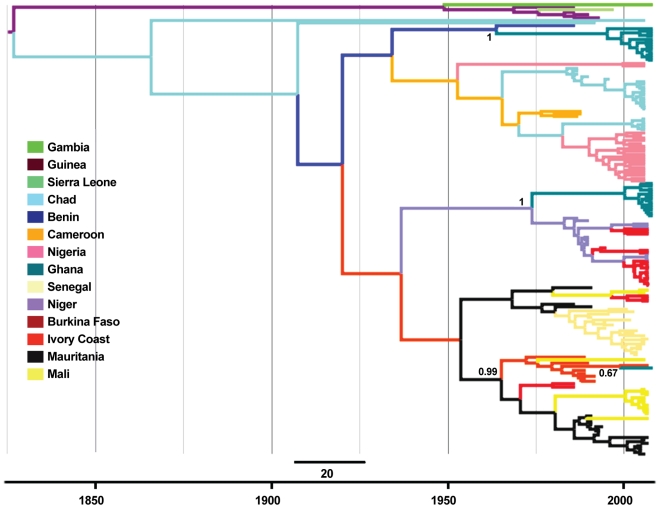
A maximum clade credibility phylogenetic tree of rabies viruses (RABV) from the Africa 2 lineage. 405base-pair regions of the RABV nucleoprotein (N) from Africa 2 lineage viruses derived from Ghanaian samples and full length RABV N sequences from pan-African countries from GenBank were analyzed. Branches are coloured according to inferred ancestral location, and posterior support for key locations discussed in the text is shown. The scale bar represents 20 years.

Within the Africa 2 lineage we detected three clades in the sample of viruses from Ghana. In order to test the hypothesis that these clades entered Ghana from different West African countries and to understand these viruses' evolutionary history, we re-analyzed the Africa 2 sequence data with 139 Africa 2 sequences alone, including the eleven used previously (Table S2, Figure 3). Thirteen Africa 2 viruses form a clade with a virus from Benin, with a time to the most recent common ancestor (TMRCA) estimated between 23 and 73 years (95% HPD) and there is a considerably higher posterior probability (0.442) for the ancestor of this clade to have originated in Benin than any other sampled country (Figure 3). A further thirteen Africa 2 viruses form a clade with viruses from Niger and Burkina Faso, with a TMRCA estimated to be between 22 and 53 years. It is most likely that this clade entered Ghana from Niger (posterior probability = 0.464). A single Africa 2 virus (G6) shares a common ancestry with viruses from Ivory Coast and Burkina Faso and has a more recent ancestry of between 1 and 20 years. There is very high support for the ancestor of this clade to have originated in the Ivory Coast, before entering Ghana.

**Figure 4:**
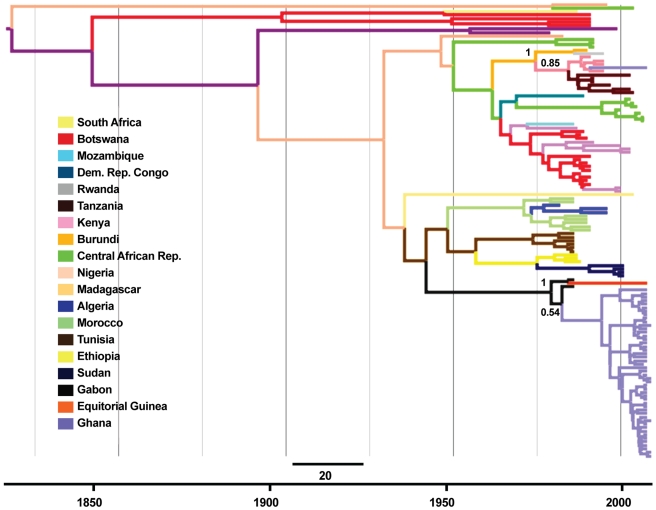
A maximum clade credibility phylogenetic tree of the Africa 1 rabies viruses (RABV) lineage. 405base-pair regions of the RABV nucleoprotein (N) from Africa 1 lineage viruses derived from Ghanaian samples and full length RABV N sequences from pan-African countries from GenBank and the VLA archive were analyzed. Branches are coloured according to inferred ancestral location, and posterior support for key locations discussed in the text is shown. The scale bar represents 20 years.

Phylogeographic analysis of the newly sequenced Ghana Africa 1 sequences with pan-African 1 sequences (Table S2, Figure 4) confirmed a monophyletic group of Africa 1a viruses (Figure 4). This clade is estimated to have emerged 23–31 years ago from Gabon (posterior probability = 0.944). The spatial analysis also provides high support for the introduction of the single Africa 1b virus from Kenya (posterior probability = 0.937) 15–22 years ago (95% HPD) (Figure 4).

**Figure 5:**
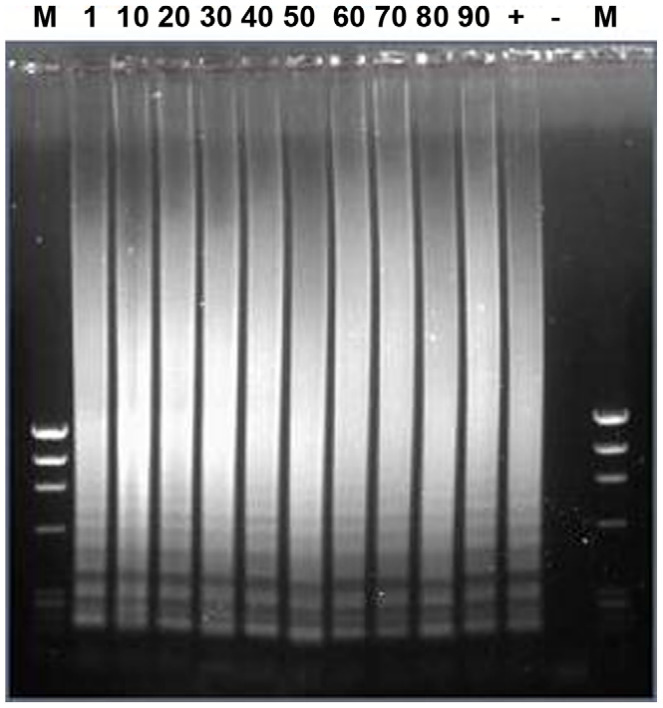
Reverse-transcription loop-mediated isothermal amplification (RT-LAMP) detection of RABV from Ghana (samples 1–90). M represents DNA markers, + is a positive control (RV202, Dog/Turkey) and − is a no-template control. Amplicons were prepared as described in Methods.

**Figure 6:**
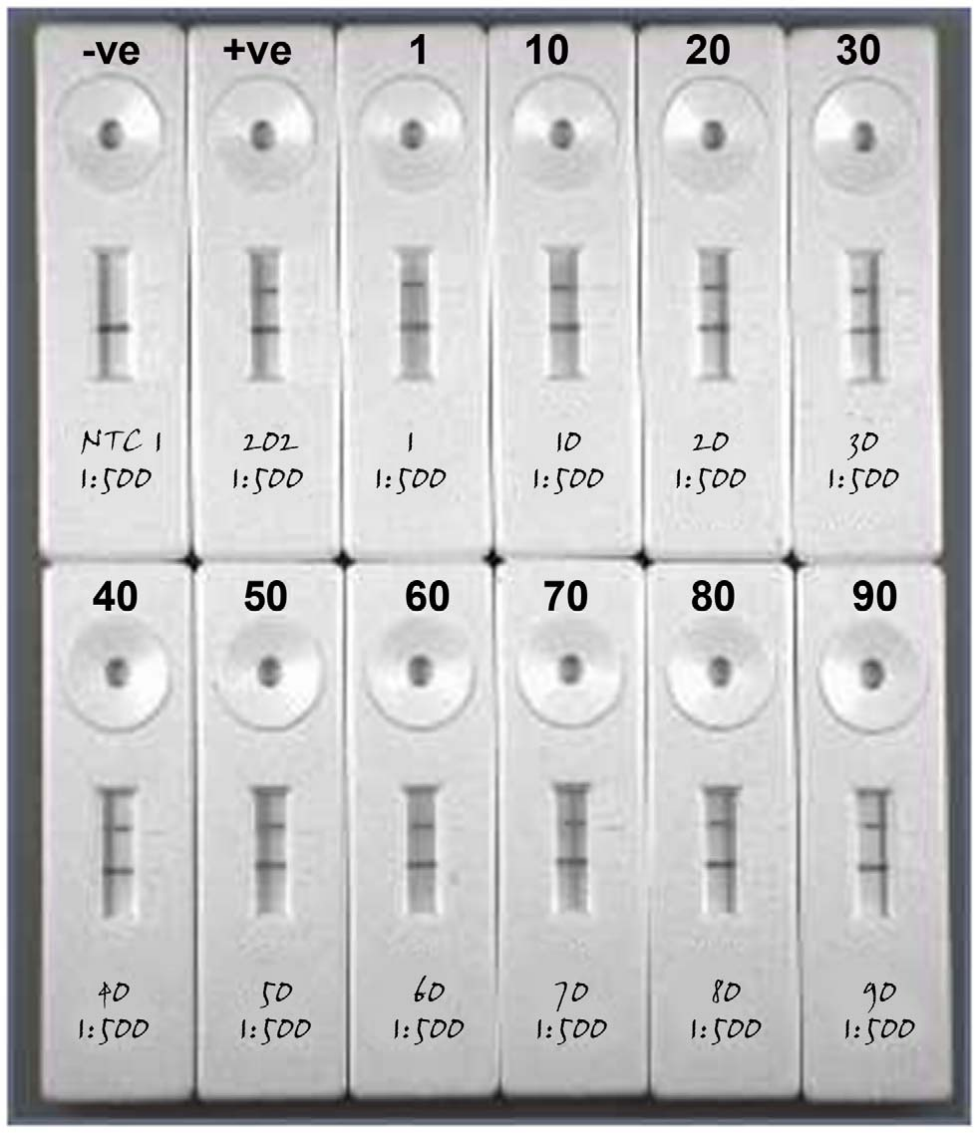
Reverse-transcription loop-mediated isothermal amplification (RT-LAMP) detection of RABV viruses from Ghana (samples 1–90). Biotinylated primers and lateral flow devices were used for product visualisation. Amplicons were prepared as described in Methods. Positive (RV202, Dog/Turkey) and negative controls are shown.

For ten randomly selected samples from the cohort, RT-LAMP detected RABV from each sample with a similar banding pattern to the positive control when separated by agarose gel electrophoresis (Figure 5) or when biotinylated products were applied to a LFD (Figure 6). This group comprised three Africa 2 and seven Africa 1 viruses (Table S1). The cost of this assay was calculated at approximately $3 per assay.

## Discussion

Each of the 76 brain samples used in this study was positive for RABV antigen. The overall topology of the phylogenetic tree produced by our analysis of the RABV N-gene sequence data available from a sample of rabid African dogs and cats in Ghana was consistent with those previously described [13], [15], [42]. This analysis of Ghanaian rabies cases is the first phylogenetic analysis of RABV from Ghana. Where this analysis is distinct from reports of RABV in other West African nations is in the diversity of viruses detected within Ghana. The samples were all taken from a relatively small geographical region with those samples not from within the greater Accra region originating from towns relatively close to Accra. These included eight viruses from Tema and five from Cape Coast (25 and 142 km from Accra, respectively). There was no evidence of infection with Africa 3 RABV (detected in mongoose in southern Africa) [22], [23], [24], Africa 4 RABV (detected in north-eastern Africa) [31] or other *Lyssavirus* species such as Lagos bat virus, against which a high seroprevalence of antibodies has been detected in bats from Accra [43]. However, our analysis suggests that rabies epidemiology is much more complex than at first thought from previous studies within West Africa. Indeed, whilst West African countries typically have defined lineages circulating within them, only Nigeria and the Central African Republic have previously been described as having Africa 1 and 2 lineage viruses co-circulating within their national borders [13], [16]. We detected both in Ghana, and propose that Ghana's recent history and geography may explain why both virus lineages were detected.

Africa 2 viruses appear to have been present within the dog populations of West Africa, including Ghana, for decades. This is derived from the close relationships between the RABV characterized in Ghana and those reported in other West African countries, such as Benin, Ivory Coast, Burkina Faso and Niger. Our results support the findings of others that the Africa 2 virus lineage has been circulating within Africa for less than 200 years [13]. Within Ghana, our analysis suggests the Africa 2 clades now co-circulating in Ghana have different evolutionary histories. From the Africa 2 phylogenetic analysis (Figure 3), we hypothesize that the three Ghanaian Africa 2 clades co-circulate in Ghana, but share evolutionary histories with viruses from other West African countries. Whilst we cannot be certain of the direction of the virus spread, we believe that there have been three different introductions of Africa 2 viruses to Ghana. We found support for the hypothesis that one clade that circulates in Ghana and in the northeasterly West African countries of Niger and Burkina Faso was originally imported from Niger and subsequently entered both Ghana and Burkina Faso (Figure 3). Another clade of viruses share a common ancestry with a Beninese isolate from the east and likely entered the country from Benin or via neighboring Togo. The evolutionary history of those viruses from the east and northeast may be due to Lake Volta providing a physical obstacle to virus transmission between dog populations. Further analysis of this phylogenetic relationship is precluded, however, by the lack of additional published sequences from Benin, and none from neighboring Togo. A single virus, G6, forms a clade with isolates from the Ivory Coast. This virus appears to be a recent introduction, sharing a TMRCA of just 1 to 20 years with viruses from the Ivory Coast to the west. A possible reason for fewer viruses being from the Ivory Coast may be the large tropical forest system along the Ghana-Ivory Coast border providing a barrier to dog movements. The border with the Ivory Coast was historically the most forested area of Ghana, however rapid deforestation and increasingly easy “between country” travel may have led to the trans-boundary movements of this virus.

Due to the historical dominance of Africa 1 viruses in the northern, eastern and southern parts of Africa, we believe it reasonable to hypothesize that Africa 1 viruses have entered Ghana from those regions, and that transmission has not been from Ghana to those regions. This hypothesis is supported by the phylogeographic analysis which suggests that the virus sub-lineage Africa 1a was transmitted from central African counties to Ghana. If we accept this, the origin of the Ghanaian Africa 1a sub-lineage viruses may be explained simply by virus transmission through dog (and potentially other vector) populations from central African nations to Ghana (Figure 4). Indeed, in our analysis the Ghanaian Africa 1a viruses share an ancestry with a virus from Gabon with a TMRCA estimated to be 23–31 years ago. This would require viruses to be transmitted at an approximate rate of between 39 to 53 kilometers per year. The large number of Africa 1a viruses in our sample suggests that this sub-lineage is well established in the Accra region, however further virus sequences from nations between Ghana and Gabon are required to confirm the evolutionary history of this sub-lineage.

The presence of an Africa 1b sub-lineage RABV in our analysis is the first reported from West Africa. Analysis of the Africa 1 lineage viruses suggests that this virus shares an ancestry with viruses from East Africa, in particular, those from Kenya (Figure 4). The presence of this virus may be explained in one of two, not exclusive, ways. Firstly, sub-lineage 1b viruses may simply have been transmitted within the populations of dogs and other susceptible animals from eastern African countries to Ghana. Transmission from Kenya (with Nairobi approximately 4200 km from Accra) would require virus transmission at a rate of approximately 190–279 kilometers per year with the TMRCA estimated to be 18 years (15–22 years, 95% HPD). Given the distance infected dogs and potential wildlife hosts may travel, this is theoretically possible, but highly unlikely given that rabies spread in red foxes and raccoons in Europe and North America was estimated to be typically 30–60 kilometers a year [44], [45]. Therefore, we hypothesize that the more likely reason for this virus' presence in Ghana is that an infected animal was translocated from the east, thus introducing a new sub-lineage to the region. Indeed, we believe that this may be the first report of molecular evidence of a long distance translocation of a rabies sub-lineage in Africa.

Spatio-temporal models of rabies in eastern and southern Africa show large-scale synchrony of rabies epidemics across both regions [46]. The analysis by Hampson *et al* provided evidence that movement of infectious animals, or animals in the incubation period, and localized regional or national vaccination campaigns during epidemics, are likely to lead to rabies synchrony [46]. However, evidence provided by rabies control programmes in both Europe and the Americas show that large-scale control programmes can be successful [47], [48], [49], [50]. A study of rabies in Tanzania also suggested dog rabies control was feasible, but was hampered by perceived problems that were largely unfounded [7]. A subsequent analysis by Hampson *et al* suggested that regular regional pulsed vaccination programmes would be required to eliminate dog rabies [51]. Despite the analysis estimating the basic reproductive rate of domestic dog rabies throughout the world to be low (R0<2), the rapid turnover of dog populations led to enough susceptible hosts for rabies to be maintained [51]. Our molecular study suggests introductions of RABV from neighboring countries into Ghana are not infrequent, demonstrating that without substantial support for continuous vaccination or coherent regional cooperation, Ghana will be unable to eliminate rabies and maintain a rabies-free status. In addition to this, our analysis provides evidence of a virus that shares a recent common ancestry with viruses from East Africa, therefore providing further evidence that regional control programmes must be implemented and that once rabies is eliminated, vigilance and technical expertise must be maintained in order for new introductions to be controlled [46].

Currently rabies diagnostics within the Ghanaian veterinary services remain limited to non-*Lyssavirus* species specific staining techniques, including the Sellers' stain and, when FITC conjugate is available, FAT. Inadequate government and financial commitments and a resource limited veterinary infrastructure are restrictive factors that preclude a sustainable rabies diagnostic service in Ghana. Surveillance activities should be given a higher priority to maintain an effective diagnostic service with the co-operation of other national and international organizations. Each of the 76 brain samples used in this study was positive for RABV infection by RT-PCR at VLA Weybridge. Of the 76 samples full histories were available for 72 positive rabies cases. However, seven samples were negative when tested by Sellers' stain at the VSL. The VSL recorded 66 humans being bitten by those 72 dogs for which histories were recorded (data not shown), including six bites to humans by the seven RABV positive cases that tested negative in the VSL. Further training and the availability of FITC conjugate for the FAT or use of the direct rapid immunohistochemical test (dRIT) [12], [52], [53] may have overcome some of the diagnostic problems. However, given that low cost isothermal RT-LAMP assays have been developed for a number of viruses affecting livestock in Africa, including Rift Valley Fever virus [33] and African Swine Fever virus [54], we developed and tested the RT-LAMP for use in African laboratories. The RT-LAMP may be prone to some of the same problems as other molecular techniques, such as cross-contamination, however it is a cheap molecular technique that produces a product that is available for further analysis such as sequencing of the approximately 200 bp product. We developed the novel RT-LAMP on randomly selected RABV samples, including both Africa 1 (a cosmopolitan) and 2 lineages. This assay successfully amplified viral genetic material producing a measurable DNA product for both Africa 1 and 2 lineage viruses. This isothermal diagnostic assay negates the need for thermal-cyclers for molecular diagnosis of RABV. The assay reagents costs approximately $3 per assay and therefore may prove a useful alternative assay for those laboratories that already have molecular expertise and adds to the range of rapid cost-effective diagnostic assays that will be fundamental if developing countries wish to develop their own RABV diagnostic capabilities. Whilst “snap test” LFD tests have previously been reported [55] our adaptation of the RT-LAMP assay to use an LFD platform, instead of UV illumination, further reduces the technology required for RABV diagnosis in African laboratories. Additional validation of this method will require comparison with the gold standard assays, assessment of larger panels of samples from throughout Africa, as well as evaluation of its sensitivity in detecting RABV in brain samples from OIE reference laboratories. These preliminary findings, however, demonstrate proof-of-concept and suggest that this technique has the potential to provide African laboratories with a cheap and rapid molecular detection method.

We conclude that our analysis of rabies virus sequences derived from Ghana has furthered the understanding of RABV epidemiology in West Africa. In particular, our analyses suggest that both Africa 1 and Africa 2 RABV lineages are present in Ghana. Africa 1b sub-lineage had previously not been reported in West Africa, and its detection, along with evidence of an additional four further clades circulating in Ghana support previous analyses that suggest that only sustained regional level approaches to rabies control will be successful in rabies elimination. In addition, we have developed an African RABV RT-LAMP assay, which can be adapted for use with LFD platforms that we advocate will provide an additional diagnostic tool for African regional laboratories.

## Supporting Information

Table S1 (0.08 MB DOCX)
