# Supplementary material for: Evolutionary History of Rabies in Ghana
Source: PLoS Negl Trop Dis. 2011 Apr 5;5(4):e1001. doi: 10.1371/journal.pntd.0001001 (PMC3071360; doi:10.1371/journal.pntd.0001001)
Supplement: Table S1 — (0.08 MB DOCX) [file pntd.0001001.s001.docx]

Supplementary Information

Table S1: Sample details reported for rabies viruses collected in Ghana and used in this study.

| **Sample No.** | **Location** | **Submission Date** | **Africa Group** | **GenBank Ref.** |
| --- | --- | --- | --- | --- |
| G1 | Accra* | 29/04/08 | 1a | HQ256770 |
| G2 | Accra* | 22/05/08 | 1a | HM368105 |
| G3 | Cape Coast | 30/04/08 | 1a | HM368113 |
| G4 | Amasaman | 26/06/08 | 1a | HM368123 |
| G5 | Accra* | 30/10/08 | 1a | HM368133 |
| G6 | Achimota | 29/01/08 | 2 | HM368142 |
| G7 | Accra* | 21/01/08 | 2 | HM368152 |
| G8 | Mallam | 20/05/08 | 1a | HM368161 |
| G9** | Cape Coast | 07/07/08 | 1a | HM368163 |
| G10 | Mallam | 20/03/08 | 2 | HM368091 |
| G11 | Cape Coast | 02/05/08 | 1a | HM368092 |
| G12 | Tema | 10/03/08 | 2 | HM368093 |
| G13 | Labadi | 13/10/08 | 1b | HM368094 |
| G15 | Tema | 24/11/08 | 2 | HM368095 |
| G16 | Accra* | 18/09/08 | 1a | HM368096 |
| G17 | Akosombo | 17/01/08 | 2 | HM368097 |
| G18 | Haatso | 10/10/08 | 2 | HM368098 |
| G20 | Dansoman | 11/02/08 | 1a | HM368099 |
| G24 | Madina | 03/01/08 | 2 | HM368100 |
| G25 | Kasoa | 02/07/08 | 1a | HM368101 |
| G27 | Accra* | 28/05/08 | 2 | HM368102 |
| G28 | Accra* | 04/11/08 | 1a | HM368103 |
| G29 | Accra* | 11/12/08 | 1a | HM368104 |
| G30 | Accra* | 21/11/08 | 1a | HM368106 |
| G32 | Abeka La-Paz | 13/05/08 | 1a | HM368107 |
| G34 | Pokuase | 25/06/08 | 1a | HM368108 |
| G35 | Cape Coast | 02/05/08 | 1a | HM368109 |
| G36 | Kofondua | 10/10/08 | 1a | HM368110 |
| G37 | Accra* | 30/10/08 | 2 | HM368111 |
| G38 | Accra* | 25/11/08 | 1a | HM368112 |
| G40 | Accra* | 28/11/08 | 1a | HM368114 |
| G41 | Brekusa | 09/10/08 | 1a | HM368115 |
| G43 | Accra* | 14/11/08 | 2 | HM368116 |
| G44 | Accra* | 25/02/08 | 1a | HM368117 |
| G45 | Somanya | 30/07/08 | 2 | HM368118 |
| G46 | Kasoa | 05/08/08 | 2 | HM368119 |
| G47 | Accra* | 14/01/08 | 1a | HM368120 |
| G48 | Accra* | 09/04/08 | 2 | HM368121 |
| G49 | Tema | 29/09/08 | 2 | HM368122 |
| G50 | Accra* | 06/11/08 | 2 | HM368124 |
| G51 | Tema | 29/09/08 | 2 | HM368125 |
| G52 | Accra* | 23/10/08 | 2 | HM368126 |
| G53 | Tema | 26/02/08 | 2 | HM368127 |
| G55 | Accra* | 01/10/08 | 1a | HM368128 |
| G56 | Accra* | 04/12/08 | 1a | HM368129 |
| G57 | Burma Camp | 25/09/08 | 1a | HM368130 |
| G58 | Ashiaman | 16/09/08 | 2 | HM368131 |
| G59 | Accra* | 28/04/08 | 2 | HM368132 |
| G60 | Saltpond | 15/08/08 | 1a | HQ256771 |
| G61 | Accra* | 18/11/08 | 1a | HM368134 |
| G62 | Accra* | 24/11/08 | 1a | HM368135 |
| G63 | Teshie | 30/09/08 | 1a | HM368136 |
| G64 | Labadi | 07/10/08 | 1a | HM368137 |
| G66 | Nii Boi Town | 05/08/08 | 1a | HM368138 |
| G67 | Ashile Botwet | 28/09/08 | 2 | HM368139 |
| G68 | Tema | 31/03/08 | 2 | HM368140 |
| G69 | Macarthy hills | 04/06/08 | 1a | HM368141 |
| G70 | Cape Coast | 02/07/08 | 1a | HM368143 |
| G71 | Burma Camp | 08/10/08 | 1a | HM368144 |
| G72 | Accra* | 30/04/08 | 1a | HM368145 |
| G74 | Accra* | 18/11/08 | 2 | HM368146 |
| G75 | Dawsoman | 25/06/08 | 1a | HM368147 |
| G76 | Accra* | 25/04/08 | 1a | HM368148 |
| G77 | Tema | 29/08/08 | 1a | HM368149 |
| G78 | Abeka La-Paz | 23/06/08 | 1a | HM368150 |
| G79 | Accra* | 28/05/08 | 2 | HM368151 |
| G80 | Juapong | 25/06/08 | 2 | HM368153 |
| G82 | Tema | 16/09/08 | 2 | HM368154 |
| G83 | Gbawe | 08/10/08 | 1a | HM368155 |
| G85 | Kokrobitey | 23/07/09 | 1a | HM368156 |
| G86 | Accra* | 29/06/09 | 1a | HM368157 |
| G87 | Accra* | 02/07/09 | 1a | HM368158 |
| G88** | Legon | 02/07/09 | 1a | HM368159 |
| G89 | Kwabenya | 13/07/09 | 1a | HM368160 |
| G90 | Dome | 19/06/09 | 1a | HQ256772 |
| G93 | Asamankese | 21/12/07 | 1a | HM368162 |

* These viruses had no specific location reported within Accra.

** These viruses were from cats

Table S2. Details of further rabies virus sequences used in this study.

| GenBank accession number | Original reference* | Origin | Year isolated | Location | Phylogenetic analysis | Reference |
| --- | --- | --- | --- | --- | --- | --- |
| EU853567 | - | Human | 1996 | Algeria | Africa 1 | Talbi et al, 2009 [13] |
| EU853568 | - | Human | 1996 | Algeria | Africa 1 | Talbi et al, 2009 [13] |
| U22643 | - | Dog | 1982 | Algeria | Overall & Africa 1 | Kissi et al, 1995[15] |
| U22485 | - | Cat | 1986 | Benin | Overall & Africa 2 | Kissi et al, 1995[15] |
| AY330739 | RV391 | Bovine | 1991 | Botswana | Africa 1 | Johnson et al 2004b [24] |
| AY330761 | RV481 | Jackal | 1991 | Botswana | Africa 1 | Johnson et al 2004b [24] |
| AY330737 | RV389 | Jackal | 1990 | Botswana | Africa 1 | Johnson et al 2004b [24] |
| AY330733 | RV385 | Jackal | 1988 | Botswana | Africa 1 | Johnson et al 2004b [24] |
| AY330738 | RV390 | Dog | 1990 | Botswana | Africa 1 | Johnson et al 2004b [24] |
| AY330740 | RV392 | Goat | 1988 | Botswana | Africa 1 | Johnson et al 2004b [24] |
| AY330741 | RV393 | Dog | 1989 | Botswana | Africa 1 | Johnson et al 2004b [24] |
| AY330763 | RV483 | Genet | 1991 | Botswana | Africa 1 | Johnson et al 2004b [24] |
| AY330764 | RV484 | Duiker | 1991 | Botswana | Africa 1 | Johnson et al 2004b [24] |
| AY330762 | RV482 | Goat | 1991 | Botswana | Africa 1 | Johnson et al 2004b [24] |
| AY330735 | - | Mongoose | 1991 | Botswana | Africa 1 | Johnson et al 2004b [24] |
| AY330752 | - | Dog | 1991 | Botswana | Africa 1 | Johnson et al 2004b [24] |
| AY330748 | - | Goat | 1988 | Botswana | Africa 1 | Johnson et al 2004b [24] |
| AY330736 | - | Bovine | 1991 | Botswana | Africa 1 | Johnson et al 2004b [24] |
| AY330734 | - | Bovine | 1990 | Botswana | Africa 1 | Johnson et al 2004b [24] |
| AY330753 | - | Dog | 1991 | Botswana | Africa 1 | Johnson et al 2004b [24] |
| U22486 | - | Dog | 1986 | Burkina Faso | Overall & Africa 2 | Kissi et al, 1995[15] |
| EU853645 | - | Dog | 1995 | Burkina Faso | Africa 2 | Talbi et al, 2009 [13] |
| EU827268 | - | Dog | 2007 | Burkina Faso | Africa 2 | Talbi et al, 2009 [13] |
| EU827269 | - | Dog | 2007 | Burkina Faso | Africa 2 | Talbi et al, 2009 [13] |
| EU827270 | - | Cat | 2007 | Burkina Faso | Africa 2 | Talbi et al, 2009 [13] |
| EU827272 | - | Dog | 2007 | Burkina Faso | Africa 2 | Talbi et al, 2009 [13] |
| EU827273 | - | Dog | 2007 | Burkina Faso | Africa 2 | Talbi et al, 2009 [13] |
| EU827274 | - | Dog | 2007 | Burkina Faso | Africa 2 | Talbi et al, 2009 [13] |
| EU827275 | - | Dog | 2007 | Burkina Faso | Africa 2 | Talbi et al, 2009 [13] |
| EU827271 | - | Dog | 2007 | Burkina Faso | Africa 2 | Talbi et al, 2009 [13] |
| EU478492 | - | Dog | 2007 | Burkina Faso | Africa 2 | De Benedictis et al, 2009 [16] |
| EU478502 | - | Dog | 2007 | Burkina Faso | Africa 2 | De Benedictis et al, 2009 [16] |
| EU478523 | - | Dog | 2007 | Burkina Faso | Africa 2 | De Benedictis et al, 2009 [16] |
| EU478519 | - | Dog | 2007 | Burkina Faso | Africa 2 | De Benedictis et al, 2009 [16] |
| EU478522 | - | Dog | 2007 | Burkina Faso | Africa 2 | De Benedictis et al, 2009 [16] |
| EU478521 | - | Dog | 2007 | Burkina Faso | Africa 2 | De Benedictis et al, 2009 [16] |
| EU478520 | - | Dog | 2007 | Burkina Faso | Africa 2 | De Benedictis et al, 2009 [16] |
| EU478524 | - | Dog | 2007 | Burkina Faso | Africa 2 | De Benedictis et al, 2009 [16] |
| EU853645 | - | Dog | 1995 | Burkina Faso | Africa 2 | Talbi et al, 2009 [13] |
| EU478518 | - | Dog | 2007 | Burkina Faso | Africa 2 | De Benedictis et al, 2009 [16] |
| EU853614 | - | Dog | 1986 | Burkina Faso | Africa 2 | Talbi et al, 2009 [13] |
| EU478515 | - | Dog | 2007 | Burkina Faso | Africa 2 | De Benedictis et al, 2009 [16] |
| EU478500 | - | Dog | 2007 | Burkina Faso | Africa 2 | De Benedictis et al, 2009 [16] |
| EU853583 | - | Jackal | 1990 | Burundi | Africa 1 | Talbi et al, 2009 [13] |
| U22634 | - | Dog | 1987 | Cameroon | Overall & Africa 2 | Kissi et al, 1995[15] |
| U22635 | - | Dog | 1988 | Cameroon | Africa 2 | Kissi et al, 1995[15] |
| U22636 | - | Dog | 1988 | Cameroon | Africa 2 | Kissi et al, 1995[15] |
| U22650 | - | Dog | 1992 | Central African Republic | Overall & Africa 1 | Kissi et al, 1995[15] |
| U22651 | - | Dog | 1992 | Central African Republic | Africa 1 | Kissi et al, 1995[15] |
| EU853586 | - | Dog | 2003 | Central African Republic | Africa 1 | Talbi et al, 2009 [13] |
| EU853590 | - | Dog | 2000 | Central African Republic | Africa 1 | Talbi et al, 2009 [13] |
| EU853651 | - | Dog | 2004 | Central African Republic | Africa 1 | Talbi et al, 2009 [13] |
| EU853584 | - | Dog | 2004 | Central African Republic | Africa 1 | Talbi et al, 2009 [13] |
| EU853585 | - | Dog | 2005 | Central African Republic | Africa 1 | Talbi et al, 2009 [13] |
| EU853587 | - | Dog | 2006 | Central African Republic | Africa 1 | Talbi et al, 2009 [13] |
| EU853588 | - | Dog | 2006 | Central African Republic | Africa 1 | Talbi et al, 2009 [13] |
| EU853589 | - | Dog | 2007 | Central African Republic | Africa 1 | Talbi et al, 2009 [13] |
| U22644 | - | Dog | 1992 | Chad | Overall & Africa 2 | Kissi et al, 1995[15] |
| EU718786 | - | Dog | 2006 | Chad | Africa 2 | Durr et al, 2008 [52] |
| EU718778 | - | Dog | 2006 | Chad | Africa 2 | Durr et al, 2008 [52] |
| EU718782 | - | Dog | 2006 | Chad | Africa 2 | Durr et al, 2008 [52] |
| EU718779 | - | Dog | 2006 | Chad | Africa 2 | Durr et al, 2008 [52] |
| EU853656 | - | Dog | 1987 | Chad | Africa 2 | Talbi et al, 2009 [13] |
| EU853655 | - | Dog | 1987 | Chad | Africa 2 | Talbi et al, 2009 [13] |
| EU853657 | - | Dog | 1987 | Chad | Africa 2 | Talbi et al, 2009 [13] |
| EU853652 | - | Dog | 1990 | Chad | Africa 2 | Talbi et al, 2009 [13] |
| EU853653 | - | Dog | 1996 | Chad | Africa 2 | Talbi et al, 2009 [13] |
| EU718735 | - | Dog | 2006 | Chad | Africa 2 | Durr et al, 2008 [52] |
| EU718771 | - | Dog | 2006 | Chad | Africa 2 | Durr et al, 2008 [52] |
| EU718736 | - | Dog | 2006 | Chad | Africa 2 | Durr et al, 2008 [52] |
| EU718783 | - | Dog | 2006 | Chad | Africa 2 | Durr et al, 2008 [52] |
| EU718766 | - | Dog | 2006 | Chad | Africa 2 | Durr et al, 2008 [52] |
| EU718763 | - | Dog | 2006 | Chad | Africa 2 | Durr et al, 2008 [52] |
| EU718785 | - | Dog | 2006 | Chad | Africa 2 | Durr et al, 2008 [52] |
| EU718742 | - | Dog | 2006 | Chad | Africa 2 | Durr et al, 2008 [52] |
| EU718762 | - | Dog | 2006 | Chad | Africa 2 | Durr et al, 2008 [52] |
| EU718759 | - | Dog | 2006 | Chad | Africa 2 | Durr et al, 2008 [52] |
| EU718740 | - | Dog | 2006 | Chad | Africa 2 | Durr et al, 2008 [52] |
| EU718765 | - | Dog | 2006 | Chad | Africa 2 | Durr et al, 2008 [52] |
| EU718777 | - | Dog | 2006 | Chad | Africa 2 | Durr et al, 2008 [52] |
| U22638 | - | Dog | 1989 | Democratic Republic of Congo | Overall & Africa 1 | Kissi et al, 1995[15] |
| U22627 | - | Human | 1979 | Egypt | Africa 1 | Kissi et al, 1995[15] |
| DQ837461 | - | Dog | 1999 | Egypt | Africa 1 | David et al, 2007 [31] |
| FJ440104 | - | Human | 2008 | Equatorial Guinea | Africa 1 | Rubin et al, 2009 [56] |
| U22637 | - | Hyena | 1987 | Ethiopia | Africa 1 | Kissi et al, 1995[15] |
| EU853581 | - | Dog | 1988 | Ethiopia | Africa 1 | Talbi et al, 2009 [13] |
| EU853580 | - | Bovine | 1987 | Ethiopia | Africa 1 | Talbi et al, 2009 [13] |
| U22630 | - | Dog | 1986 | Gabon | Overall & Africa 1 | Kissi et al, 1995[15] |
| U22629 | - | Dog | 1986 | Gabon | Africa 1 | Kissi et al, 1995[15] |
| EU853591 | - | Dog | 2008 | Gambia | Africa 2 | Talbi et al, 2009 [13] |
| U22641 | - | Dog | 1990 | Guinea | Overall & Africa 2 | Kissi et al, 1995[15] |
| U22487 | - | Dog | 1986 | Guinea | Overall & Africa 2 | Kissi et al, 1995[15] |
| EU853594 | - | Dog | 1993 | Guinea | Africa 2 | Talbi et al, 2009 [13] |
| EU853593 | - | Dog | 1986 | Guinea | Africa 2 | Talbi et al, 2009 [13] |
| U22639 | - | Dog | 1989 | Ivory Coast | Overall & Africa 2 | Kissi et al, 1995[15] |
| U22646 | - | Dog | 1990 | Ivory Coast | Overall & Africa 2 | Kissi et al, 1995[15] |
| EU853615 | - | Dog | 2007 | Ivory Coast | Overall & Africa 2 | Talbi et al, 2009 [13] |
| EU853616 | - | Dog | 1989 | Ivory Coast | Overall & Africa 2 | Talbi et al, 2009 [13] |
| EU853618 | - | Dog | 1992 | Ivory Coast | Africa 2 | Talbi et al, 2009 [13] |
| EU853619 | - | Dog | 1992 | Ivory Coast | Africa 2 | Talbi et al, 2009 [13] |
| EU853617 | - | Dog | 1989 | Ivory Coast | Africa 2 | Talbi et al, 2009 [13] |
| EU853621 | - | Dog | 1989 | Ivory Coast | Africa 2 | Talbi et al, 2009 [13] |
| EU853618 | - | Dog | 1992 | Ivory Coast | Africa 2 | Talbi et al, 2009 [13] |
| AY502137 | RV749 | Unknown | 1995 | Kenya | Africa 1 | Johnson et al, 2004a [57] |
| AY103013 | RV460 | Dog | 1994 | Kenya | Africa 1 | Johnson et al, 2004a [57] |
| AY502136 | RV748 | Unknown | 1995 | Kenya | Africa 1 | Johnson et al, 2004a [57] |
| AY502138 | RV754 | Unknown | 1995 | Kenya | Africa 1 | Johnson et al, 2004a [57] |
| DQ420623 | - | Human | 2004 | Madagascar | Africa 1 | Iehle et al, 2008 [58] |
| EU853623 | - | Dog | 2007 | Mali | Africa 2 | Talbi et al, 2009 [13] |
| EU853622 | - | Dog | 2006 | Mali | Africa 2 | Talbi et al, 2009 [13] |
| EU853620 | - | Dog | 2006 | Mali | Africa 2 | Talbi et al, 2009 [13] |
| EU853612 | - | Dog | 2007 | Mali | Africa 2 | Talbi et al, 2009 [13] |
| U22649 | - | Jackal | 1992 | Namibia | Overall & Africa 1 | Kissi et al, 1995[15] |
| EU853601 | - | Dog | 2006 | Mali | Africa 2 | Talbi et al, 2009 [13] |
| EU853598 | - | Dog | 2006 | Mali | Africa 2 | Talbi et al, 2009 [13] |
| EU853602 | - | Dog | 2006 | Mali | Africa 2 | Talbi et al, 2009 [13] |
| EU853605 | - | Dog | 2006 | Mali | Africa 2 | Talbi et al, 2009 [13] |
| EU853612 | - | Dog | 2007 | Mali | Africa 2 | Talbi et al, 2009 [13] |
| EU853599 | - | Dog | 2007 | Mali | Africa 2 | Talbi et al, 2009 [13] |
| EU853597 | - | Dog | 2007 | Mali | Africa 2 | Talbi et al, 2009 [13] |
| EU853603 | - | Dog | 2007 | Mali | Africa 2 | Talbi et al, 2009 [13] |
| EU853596 | - | Dog | 2007 | Mali | Africa 2 | Talbi et al, 2009 [13] |
| EU853604 | - | Dog | 2007 | Mali | Africa 2 | Talbi et al, 2009 [13] |
| EU853600 | - | Dog | 2007 | Mali | Africa 2 | Talbi et al, 2009 [13] |
| DQ194864 | RV1497 | Jackal | 2000 | Namibia | Africa 1 | Mansfield et al, 2006 [28] |
| DQ194865 | RV1498 | Jackal | 2000 | Namibia | Africa 1 | Mansfield et al, 2006 [28] |
| DQ194866 | RV1499 | Jackal | 2000 | Namibia | Africa 1 | Mansfield et al, 2006 [28] |
| DQ194861 | RV1493 | Kudu | 2003 | Namibia | Africa 1 | Mansfield et al, 2006 [28] |
| DQ194863 | RV1494 | Kudu | 2003 | Namibia | Africa 1 | Mansfield et al, 2006 [28] |
| U22632 | - | Kudu | 1987 | Namibia | Africa 1 | Kissi et al, 1995[15] |
| U22489 | - | Camel | 1986 | Mauritania | Africa 1 | Kissi et al, 1995[15] |
| EU853624 | - | Camel | 1991 | Mauritania | Africa 1 | Talbi et al, 2009 [13] |
| EU853642 | - | Goat | 1991 | Mauritania | Africa 1 | Talbi et al, 2009 [13] |
| EU853613 | - | Donkey | 1991 | Mauritania | Africa 1 | Talbi et al, 2009 [13] |
| EU853607 | - | Dog | 1991 | Mauritania | Africa 1 | Talbi et al, 2009 [13] |
| EU853608 | - | Dog | 1991 | Mauritania | Africa 1 | Talbi et al, 2009 [13] |
| EU853611 | - | Dog | 1994 | Mauritania | Africa 1 | Talbi et al, 2009 [13] |
| EU853610 | - | Dog | 1993 | Mauritania | Africa 1 | Talbi et al, 2009 [13] |
| EU853609 | - | Dog | 1993 | Mauritania | Africa 1 | Talbi et al, 2009 [13] |
| EU853606 | - | Dog | 1993 | Mauritania | Africa 1 | Talbi et al, 2009 [13] |
| EU514581 | - | Goat | 2007 | Mauritania | Africa 1 | Talbi et al, 2009 [13] |
| EU514578 | - | Dog | 2006 | Mauritania | Africa 1 | Talbi et al, 2009 [13] |
| EU514576 | - | Dog | 2005 | Mauritania | Africa 1 | Talbi et al, 2009 [13] |
| EU514577 | - | Dog | 2005 | Mauritania | Africa 1 | Talbi et al, 2009 [13] |
| EU514575 | - | Dog | 2005 | Mauritania | Africa 1 | Talbi et al, 2009 [13] |
| EU514580 | - | Dog | 2006 | Mauritania | Africa 1 | Talbi et al, 2009 [13] |
| U22631 | - | Dog | 1986 | Morocco | Overall & Africa 1 | Kissi et al, 1995[15] |
| EU853572 | - | Human | 1986 | Morocco | Africa 1 | Talbi et al, 2009 [13] |
| EU853571 | - | Human | 1991 | Morocco | Africa 1 | Talbi et al, 2009 [13] |
| EU853569 | - | Dog | 1990 | Morocco | Africa 1 | Talbi et al, 2009 [13] |
| EU853570 | - | Human | 1991 | Morocco | Africa 1 | Talbi et al, 2009 [13] |
| U22852 | - | Human | 1990 | Morocco | Africa 1 | Kissi et al, 1995[15] |
| U22642 | - | Fox | 1990 | Morocco | Africa 1 | Kissi et al, 1995[15] |
| U22484 | - | Dog | 1986 | Mozambique | Africa 1 | Kissi et al, 1995[15] |
| U22640 | - | Dog | 1990 | Niger | Overall & Africa 2 | Kissi et al, 1995[15] |
| EU853646 | - | Dog | 1990 | Niger | Africa 2 | Talbi et al, 2009 [13] |
| EU853647 | - | Dog | 1990 | Niger | Africa 2 | Talbi et al, 2009 [13] |
| EU853649 | - | Dog | 1990 | Niger | Africa 2 | Talbi et al, 2009 [13] |
| EU853648 | - | Dog | 1990 | Niger | Africa 2 | Talbi et al, 2009 [13] |
| EU514573 | - | Dog | 2007 | Niger | Africa 2 | Talbi et al, 2009 [13] |
| EU514574 | - | Dog | 2007 | Niger | Africa 2 | Talbi et al, 2009 [13] |
| EU514572 | - | Dog | 2007 | Niger | Africa 2 | Talbi et al, 2009 [13] |
| EU514571 | - | Dog | 2007 | Niger | Africa 2 | Talbi et al, 2009 [13] |
| EU853650 | - | Dog | 1987 | Niger | Africa 2 | Talbi et al, 2009 [13] |
| U22488 | - | Human | 1983 | Nigeria | Overall & Africa 1 | Kissi et al, 1995[15] |
| AY103008 | RV629 | Dog | 1996 | Nigeria | Africa 1 | Johnson et al, 2004a [57] |
| EU038091 | - | Dog | 2005 | Nigeria | Africa 2 | - |
| EU038092 | - | Dog | 2006 | Nigeria | Africa 2 | - |
| EU038093 | - | Dog | 2005 | Nigeria | Africa 2 | - |
| EU038094 | - | Dog | 2005 | Nigeria | Africa 2 | - |
| EU038096 | - | Dog | 2006 | Nigeria | Africa 2 | - |
| EU038097 | - | Dog | 2006 | Nigeria | Africa 2 | - |
| EU038090 | - | Dog | 2005 | Nigeria | Africa 2 | - |
| EU038099 | - | Dog | 2006 | Nigeria | Africa 2 | - |
| EU038089 | - | Dog | 2005 | Nigeria | Africa 2 | - |
| EU038095 | - | Dog | 2006 | Nigeria | Africa 2 | - |
| EU038082 | - | Dog | 2006 | Nigeria | Africa 2 | - |
| EU038101 | - | Dog | 2006 | Nigeria | Africa 2 | - |
| EU038102 | - | Dog | 2006 | Nigeria | Africa 2 | - |
| EU038103 | - | Dog | 2006 | Nigeria | Africa 2 | - |
| EU038105 | - | Dog | 2006 | Nigeria | Africa 2 | - |
| EU038106 | - | Dog | 2006 | Nigeria | Africa 2 | - |
| EU038107 | - | Dog | 2006 | Nigeria | Africa 2 | - |
| EU038108 | - | Dog | 2006 | Nigeria | Africa 2 | - |
| EU038109 | - | Dog | 2006 | Nigeria | Africa 2 | - |
| EU038098 | - | Dog | 2006 | Nigeria | Africa 2 | - |
| EU038104 | - | Dog | 2006 | Nigeria | Africa 2 | - |
| EU853582 | - | Dog | 1995 | Rwanda | Africa 1 | Talbi et al, 2009 [13] |
| EU853631 | - | Human | 2005 | Senegal | Africa 2 | Talbi et al, 2009 [13] |
| EU853632 | - | Human | 2005 | Senegal | Africa 2 | Talbi et al, 2009 [13] |
| EU853633 | - | Human | 2004 | Senegal | Africa 2 | Talbi et al, 2009 [13] |
| EU853634 | - | Dog | 2004 | Senegal | Africa 2 | Talbi et al, 2009 [13] |
| EU853635 | - | Human | 2006 | Senegal | Africa 2 | Talbi et al, 2009 [13] |
| EU853636 | - | Human | 2006 | Senegal | Africa 2 | Talbi et al, 2009 [13] |
| EU853637 | - | Human | 2003 | Senegal | Africa 2 | Talbi et al, 2009 [13] |
| EU853638 | - | Human | 1995 | Senegal | Africa 2 | Talbi et al, 2009 [13] |
| EU853639 | - | Human | 2004 | Senegal | Africa 2 | Talbi et al, 2009 [13] |
| EU853640 | - | Human | 1996 | Senegal | Africa 2 | Talbi et al, 2009 [13] |
| EU853641 | - | Dog | 1991 | Senegal | Africa 2 | Talbi et al, 2009 [13] |
| EU853643 | - | Human | 2003 | Senegal | Africa 2 | Talbi et al, 2009 [13] |
| EU853644 | - | Human | 2001 | Senegal | Africa 2 | Talbi et al, 2009 [13] |
| EU853625 | - | Dog | 1995 | Senegal | Africa 2 | Talbi et al, 2009 [13] |
| EU853626 | - | Human | 1997 | Senegal | Africa 2 | Talbi et al, 2009 [13] |
| EU853627 | - | Human | 2002 | Senegal | Africa 2 | Talbi et al, 2009 [13] |
| EU853628 | - | Human | 2007 | Senegal | Africa 2 | Talbi et al, 2009 [13] |
| EU853629 | - | Cat | 2006 | Senegal | Africa 2 | Talbi et al, 2009 [13] |
| EU853595 | - | Cat | 1997 | Sierra Leone | Africa 2 | Talbi et al, 2009 [13] |
| U22628 | - | Mongoose | 1987 | South Africa | Overall & Africa 1 | Kissi et al, 1995[15] |
| AY502126 | RV1346 | Dog | 2001 | Sudan | Africa 1 | Johnson et al, 2004a [57] |
| AY502127 | RV1352 | Dog | 2001 | Sudan | Africa 1 | Johnson et al, 2004a [57] |
| AY502125 | RV1342 | Dog | 2001 | Sudan | Africa 1 | Johnson et al, 2004a [57] |
| AY502128 | RV1360 | Dog | 2001 | Sudan | Africa 1 | Johnson et al, 2004a [57] |
| U22645 | - | Dog | 1992 | Tanzania | Overall & Africa 1 | Kissi et al, 1995[15] |
| U22647 | - | Cow | 1992 | Tanzania | Africa 1 | Kissi et al, 1995[15] |
| HQ660809 | RV1012 | Dog | 2000 | Tanzania | Africa 1 | Lembo et al, 2007 [18]** |
| HQ660808 | RV1011 | Dog | 2000 | Tanzania | Africa 1 | Lembo et al, 2007 [18]** |
| DQ900555 | RV1013 | Dog | 2000 | Tanzania | Africa 1 | Lembo et al, 2007 [18] |
| DQ900556 | - | Dog | 2003 | Tanzania | Africa 1 | Lembo et al, 2007 [18] |
| DQ900559 | - | Goat | 1997 | Tanzania | Africa 1 | Lembo et al, 2007 [18] |
| DQ900569 | - | Hyena | 2004 | Tanzania | Africa 1 | Lembo et al, 2007 [18] |
| DQ900570 | - | Genet | 2003 | Tanzania | Africa 1 | Lembo et al, 2007 [18] |
| EU853579 | - | Human | 1986 | Tunisia | Africa 1 | Talbi et al, 2009 [13] |
| EU853578 | - | Human | 1986 | Tunisia | Africa 1 | Talbi et al, 2009 [13] |
| EU853577 | - | Human | 1986 | Tunisia | Africa 1 | Talbi et al, 2009 [13] |
| EU853576 | - | Human | 1986 | Tunisia | Africa 1 | Talbi et al, 2009 [13] |
| EU853575 | - | Human | 1986 | Tunisia | Africa 1 | Talbi et al, 2009 [13] |
| EU853574 | - | Human | 1986 | Tunisia | Africa 1 | Talbi et al, 2009 [13] |

* 405 nucleotide (nt) regions from the VLA archive were used for this analysis.

** 405 nucleotide (nt) regions from the VLA archive were used for this analysis and the updated sequences submitted to GenBank.

References

57. Johnson N, McElhinney LM, Ali YH, Saeed IK, Fooks AR (2004) Molecular epidemiology of canid rabies in Sudan: evidence for a common origin of rabies with Ethiopia. Virus Res 104: 201-205.

58. Iehle C, Dacheux L, Ralandison S, Rakoto Andrianarivelo M, Rousset D, et al. (2008) Delivery and follow-up of a healthy newborn from a mother with clinical rabies. J Clin Virol 42: 82-85.
